# Incidental Amyand’s Hernia During Enhanced-View Totally Extraperitoneal (eTEP) Repair of Recurrent Inguinal Hernia: A Case Report and Technical Rationale for a Posterior Approach

**DOI:** 10.7759/cureus.105963

**Published:** 2026-03-27

**Authors:** Ender Bademkiran

**Affiliations:** 1 Department of General Surgery, Akhisar Mustafa Kirazoğlu State Hospital, Manisa, TUR

**Keywords:** amyand’s hernia, etep repair, laparoscopic hernia repair, minimally invasive surgery, recurrent inguinal hernia

## Abstract

Incidental Amyand’s hernia is a rare intraoperative finding during inguinal hernia repair and may pose a management dilemma, particularly in recurrent cases where prior anterior repair limits surgical options. In these settings, careful consideration is required for optimal plane selection and the safety of mesh placement after appendectomy.

A 67-year-old man with a history of prior anterior Lichtenstein repair presented with a recurrent right inguinal hernia. A posterior laparo-endoscopic repair using the enhanced-view totally extraperitoneal (eTEP) technique was performed in accordance with international guidelines. During hernia sac dissection, a controlled peritoneal opening was intentionally created to safely identify the sac contents. The cecum and a macroscopically normal appendix were found within the sac, consistent with a Type I Amyand’s hernia. Laparoscopic appendectomy was performed, and the repair was completed in the preperitoneal space using the eTEP approach, without conversion to a transabdominal preperitoneal (TAPP) technique. The postoperative course was uneventful, with no recurrence or chronic pain at six-month follow-up.

This case demonstrates the utility of the eTEP approach as a posterior strategy for recurrent inguinal hernia repair when unexpected intraoperative findings occur. In selected patients with non-inflamed Amyand’s hernia, appendectomy combined with prosthetic mesh repair can be performed safely without compromising outcomes.

## Introduction

Amyand’s hernia is an uncommon intraoperative finding encountered during inguinal hernia repair and is defined by the presence of the vermiform appendix within the hernia sac. Although rare, its management remains controversial, as operative decisions depend on the status of the appendix, the risk of contamination, and the chosen hernia repair technique [1,2].

Preoperative diagnosis is uncommon, particularly in non-inflamed cases, and the condition is most often identified incidentally during surgery [3]. This scenario can create a complex intraoperative dilemma, especially in recurrent inguinal hernias, where prior anterior repair may limit safe dissection and influence the choice of surgical plane [4]. Amyand’s hernia presents unique challenges in minimally invasive approaches, where both the operative plane and management of unexpected findings require careful intraoperative judgment.

Current international groin hernia guidelines recommend posterior laparo-endoscopic approaches for recurrent hernias following anterior repair, as these techniques utilize an unviolated anatomical plane. The enhanced-view totally extraperitoneal (eTEP) approach expands the retromuscular working space and may offer technical advantages when unexpected intraoperative findings are encountered [5].

Herein, we present a case of an incidentally discovered non-inflamed Amyand’s hernia during eTEP repair of a recurrent inguinal hernia. This case highlights the rarity of such findings during eTEP repair and demonstrates that unexpected intra-sac pathology can be safely managed without conversion from a posterior minimally invasive approach.

## Case presentation

In September 2025, a 67-year-old man presented to the Department of General Surgery at Akhisar Mustafa Kirazoğlu State Hospital, Manisa, Turkey, with a two-year history of progressive right groin swelling and intermittent discomfort. He had previously undergone an open anterior Lichtenstein repair 34 years earlier. Physical examination revealed a reducible recurrent right inguinal hernia without signs of incarceration or bowel obstruction. Preoperative ultrasonography demonstrated an approximately 3-4 cm fascial defect; however, the contents of the hernia sac could not be clearly delineated.

Given the history of prior anterior repair, a posterior laparo-endoscopic approach was selected in accordance with international groin hernia guidelines to avoid dissection in scarred anterior tissues and to utilize an unviolated preperitoneal plane.

**Figure 1:**
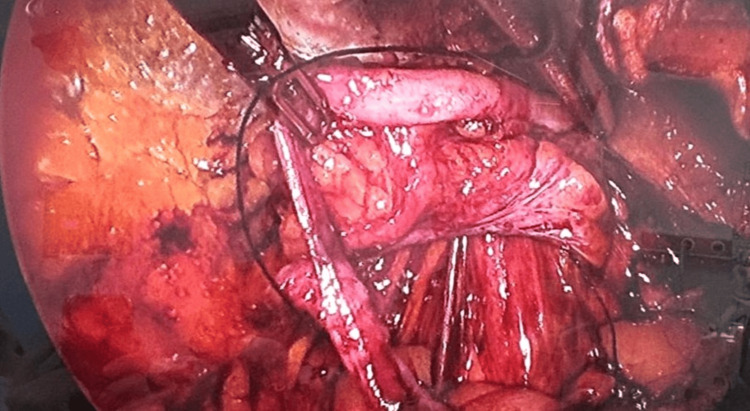
Intraoperative view demonstrating a macroscopically normal vermiform appendix within the indirect recurrent inguinal hernia sac, consistent with Type I Amyand’s hernia.

Under general anesthesia, an eTEP repair was performed. A retromuscular working space was established, followed by careful development of the preperitoneal plane. Dissection toward the myopectineal orifice revealed an indirect hernia sac measuring approximately 3-4 cm, containing both the cecum and appendix. Significant resistance due to adhesions from the previous anterior repair necessitated a controlled and intentional peritoneal opening to allow safe identification of the sac contents. Inspection demonstrated a macroscopically normal appendix within the sac, consistent with a Type I Amyand’s hernia (Figure 1).

No additional intraperitoneal ports were placed. Pneumoperitoneum was temporarily established through the controlled peritoneal opening to facilitate appendectomy. Following completion of the appendectomy, the procedure was continued in the extraperitoneal plane without creation of a peritoneal flap. Given the recurrent setting and the potential risk of future diagnostic confusion or reoperation within a previously repaired groin, a laparoscopic appendectomy was performed through the limited peritoneal opening. Importantly, this did not represent conversion to a transabdominal preperitoneal (TAPP) approach, as the preperitoneal dissection plane was preserved and the repair was completed using the eTEP technique. The appendiceal base was secured using a standard laparoscopic method, and the operative field was irrigated with no evidence of contamination.

**Figure 2:**
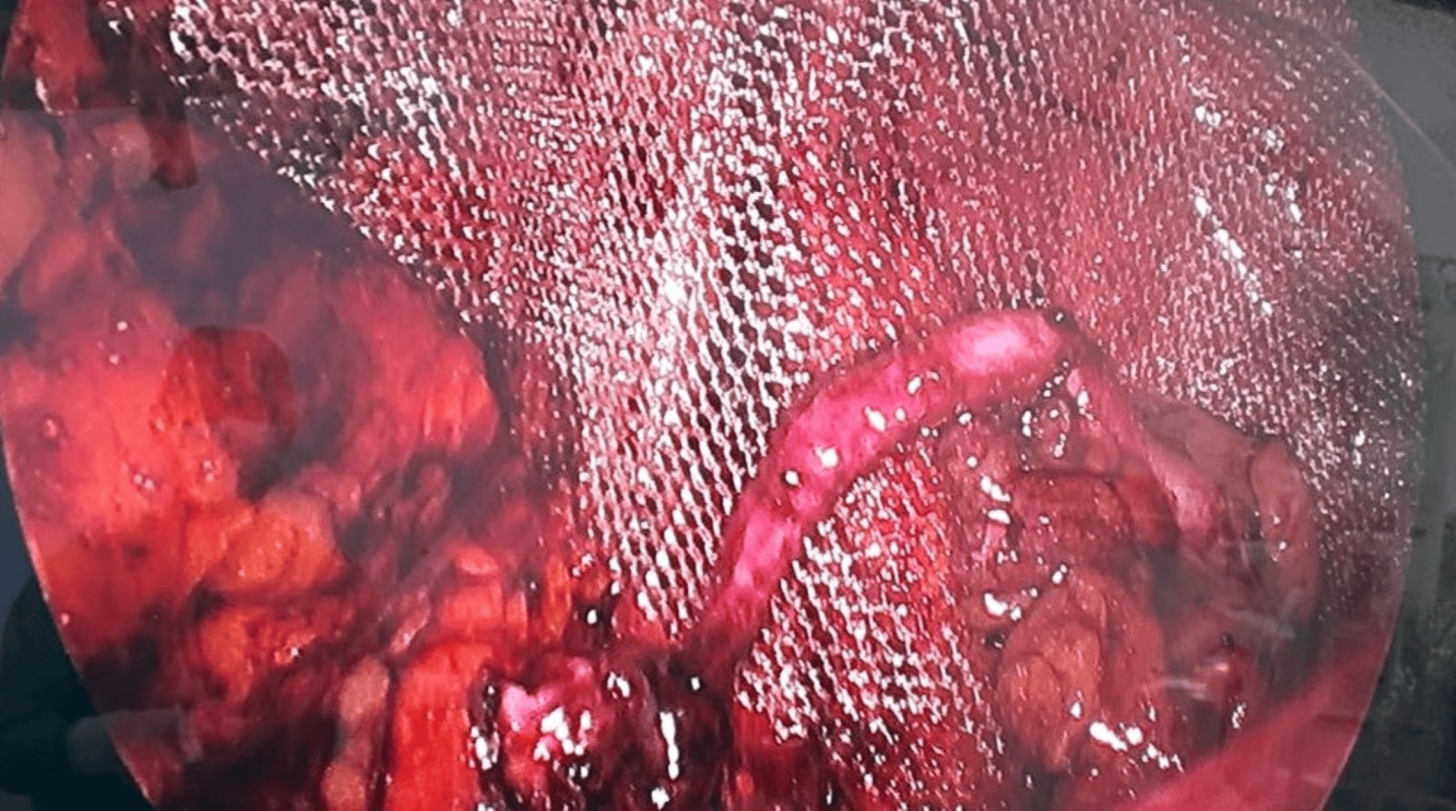
Posterior preperitoneal placement of macroporous polypropylene mesh after reduction of the appendix using the eTEP approach, demonstrating complete coverage of the myopectineal orifice. eTEP: enhanced-view totally extraperitoneal.

Following appendectomy, preperitoneal dissection was completed. A 15 × 10 cm macroporous monofilament polypropylene mesh was placed to ensure adequate coverage of the myopectineal orifice and secured using absorbable fixation devices (Figure 2). The peritoneum was restored, and the procedure was completed without intraoperative complications.

The total operative time was 45 minutes. The patient resumed oral intake on postoperative day 1 and was discharged on postoperative day 2. At the six-month follow-up, there was no evidence of recurrence, chronic postoperative inguinal pain, surgical site infection, or mesh-related complications. The wide retromuscular exposure provided by the eTEP approach facilitated controlled management of the unexpected hernia sac contents and allowed safe completion of a definitive posterior mesh repair.

## Discussion

Amyand’s hernia is most often diagnosed intraoperatively, as clinical and radiological findings are typically nonspecific, particularly in the absence of appendiceal inflammation [6]. The condition represents a spectrum ranging from an incidental finding of a normal appendix to more complex presentations involving appendicitis, perforation, or abdominal sepsis [7].

The widely accepted Losanoff and Basson classification provides a practical framework for management by correlating appendiceal status with operative strategy. Type I Amyand’s hernia describes the presence of a normal appendix within the hernia sac and generally permits mesh-based hernia repair, whereas inflamed or perforated appendices require modification of the surgical approach and careful consideration of contamination risk [8]. The present case corresponds to a Type I Amyand’s hernia, supporting the feasibility of appendectomy combined with prosthetic repair in a clean surgical field [9].

Management of a non-inflamed appendix in Amyand’s hernia remains controversial. Some authors advocate simple reduction without appendectomy to avoid unnecessary contamination, whereas others recommend prophylactic appendectomy to prevent future diagnostic uncertainty [10,11]. In the present case, appendectomy was favored given the recurrent nature of the hernia following prior anterior repair. Removal of the appendix eliminated the risk of future appendiceal pathology within a previously operated groin and reduced the likelihood of requiring reoperation in scarred tissues [12].

Recurrent inguinal hernias present a distinct technical challenge. International groin hernia guidelines recommend a posterior laparo-endoscopic approach after failed anterior repair, as this strategy utilizes an unviolated anatomical plane and minimizes dissection through fibrotic tissue [13]. Additionally, the posterior approach allows comprehensive visualization of the myopectineal orifice, facilitating identification of unexpected hernia sac contents [14].

**Figure 3:**
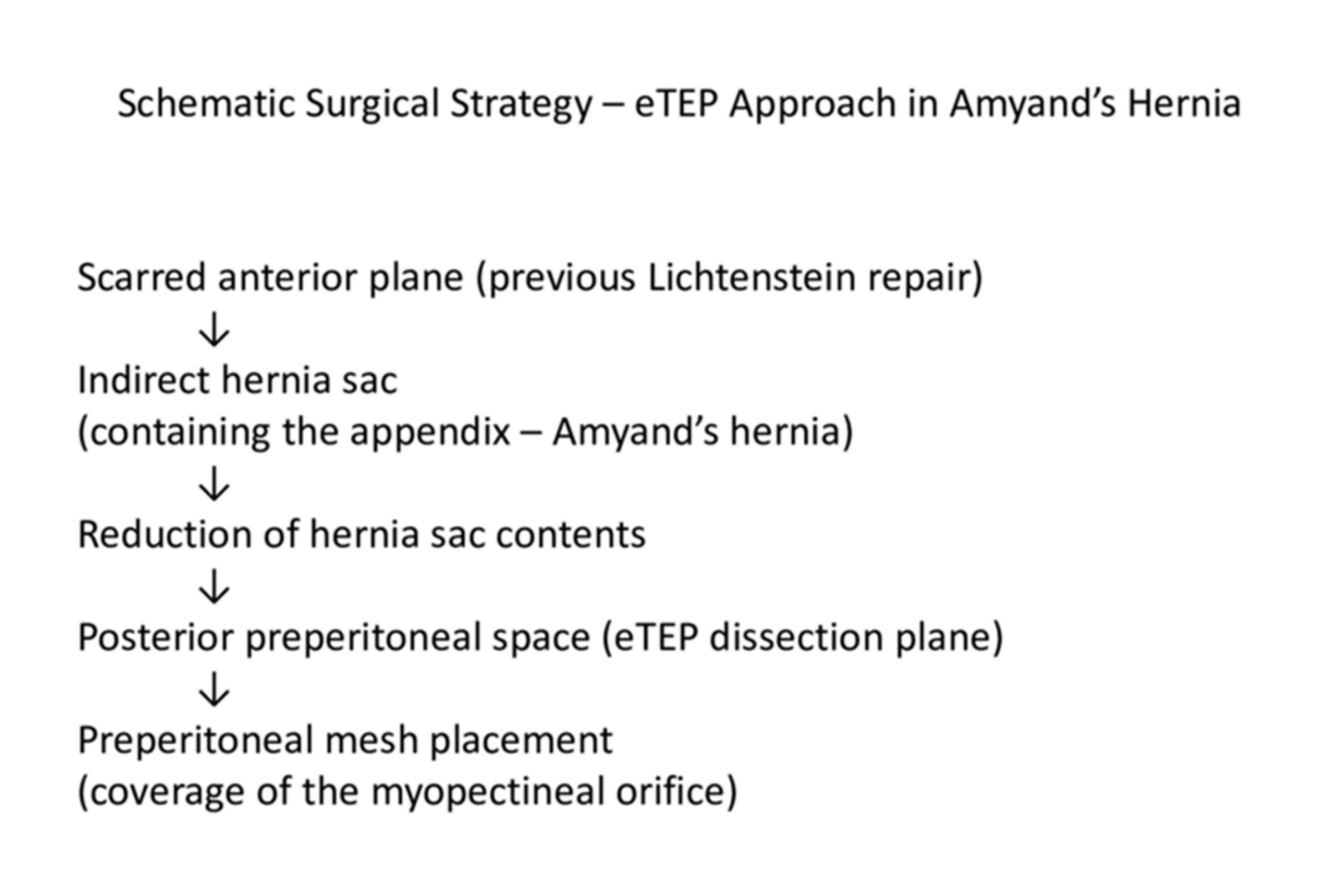
Schematic illustration of the posterior eTEP approach in recurrent inguinal hernia with an incidental Amyand’s hernia. The diagram demonstrates the posterior preperitoneal dissection plane used to avoid scarred anterior tissues, allowing safe reduction of the appendix and definitive mesh placement. This schematic diagram was created using Microsoft PowerPoint (Microsoft Corp., Redmond, WA, USA). eTEP: enhanced-view totally extraperitoneal.

The enhanced-view totally extraperitoneal (eTEP) technique further expands the retromuscular workspace compared with conventional totally extraperitoneal repair, improving ergonomics, instrument triangulation, and anatomical orientation. The conceptual surgical strategy-selecting a posterior preperitoneal plane to avoid scarred anterior tissues while enabling safe reduction and mesh placement-is illustrated schematically in Figure 3. In this case, the wide posterior exposure afforded by eTEP enabled controlled reduction of the hernia sac, safe inspection of intra-abdominal structures, and completion of definitive repair without conversion to an anterior approach [15]. Unlike the transabdominal preperitoneal (TAPP) technique, in which the peritoneal cavity is intentionally entered at the outset, this case was managed using a primarily extraperitoneal strategy. The peritoneal cavity was accessed in a limited, situation-driven manner to facilitate safe identification and management of the hernia contents, without altering the overall eTEP-based repair concept.

An additional consideration is mesh implantation following appendectomy. Although prosthetic repair has traditionally been avoided in potentially contaminated fields, accumulating evidence suggests that synthetic mesh placement is safe in the absence of active infection or perforation. In this case, careful intraoperative assessment confirmed a clean field, allowing tension-free mesh repair without postoperative complications.

This case also demonstrates that an intraoperative peritoneal breach during eTEP does not necessarily constitute conversion to a TAPP approach, provided that the dissection plane and mesh placement remain within the preperitoneal space.

An important surgical principle is highlighted: unexpected intraoperative findings during recurrent hernia repair do not mandate abandonment of a minimally invasive posterior strategy. Rather, adequate posterior exposure may offer superior control, enabling simultaneous management of incidental pathology while preserving optimal conditions for durable mesh repair.

To our knowledge, reports specifically describing incidental Amyand’s hernia managed during eTEP repair in a recurrent inguinal setting remain limited. This case was reported in accordance with the CARE guidelines.

Taken together, these findings demonstrate that the posterior eTEP approach can serve as a reliable problem-solving platform when unexpected intraoperative findings arise during recurrent inguinal hernia repair, consistent with previously published literature [1-15].

## Conclusions

Incidental Amyand’s hernia may be encountered during minimally invasive repair of recurrent inguinal hernias and may present an unexpected intraoperative challenge. The posterior laparo-endoscopic approach using the eTEP technique provides wide preperitoneal exposure, facilitating safe reduction of unusual hernia sac contents while allowing definitive mesh repair within an unviolated anatomical plane. In selected patients with a non-inflamed appendix, appendectomy combined with posterior prosthetic mesh repair appears feasible and safe. This case highlights the value of posterior surgical strategies as a problem-solving platform in complex reoperative groin hernia surgery.
